# Patients Lost to Follow-Up for Cervical Cancer in the Limbe Regional Hospital

**DOI:** 10.1200/JGO.18.00067

**Published:** 2019-02-01

**Authors:** Robert Tchounzou, André Gaetan Simo Wambo, Théophile Nana Njamen, Ingrid Ofakem Ilick, Humphry Tatah Neng, François Dadao, Albert Mouelle Sone

**Affiliations:** ^1^ Regional Hospital Limbe, Limbe, Cameroon; ^2^ University of Buea, Buea, Cameroon; ^3^ Douala General Hospital, Douala, Cameroon; ^4^ Gynaeco-Obstetric and Paediatric Hospital, Douala, Cameroon; ^5^ University of Douala, Douala, Cameroon

## Abstract

**PURPOSE:**

Cervical cancer constitutes a public health problem in Cameroon where it represents 13.8% of cancers in women. We wanted to evaluate compliance with cervical cancer care with a focus on patients who are lost to follow-up from the time that symptoms suggestive of cervical cancer are clinically recognized to treatment.

**PATIENTS AND METHODS:**

Sociodemographic data, attitude toward diagnosis and treatment, and reason for discontinuing care were recorded and analyzed for a period of 5 years from January 2010 to December 2015.

**RESULTS:**

One hundred twenty-six patients had symptoms suggestive of cervical cancer, but only 110 (87.30%) could pay for biopsy, 29 (26.36%) of those did not collect their results, 17 (18.7%) denied their results, and 20 (19%) did not benefit from treatment. Only 44 of 110 patients were able to finish their cancer care treatment program. Reasons for discontinuing the cancer care included lack of financial means to pay for it, distance from the care center, and belief in alternative treatments.

**CONCLUSION:**

This study highlights the magnitude of the difficulties of accessing and receiving cancer care in semiurban areas in Cameroon. Poverty, belief in alternative treatment options, and unequal distribution of care services determined which patients would be lost to follow-up. Redistribution of resources and cancer care providers is mandatory to improve this situation.

## INTRODUCTION

The burden of noncommunicable diseases in general and cancer in particular is increasing in Cameroon because of changes in lifestyle and massive urbanization of the population.^1^ In 2008, the International Agency for Research on Cancer estimated that population-based, age-standardized incidence of cancers for both sexes was 92.1 per 100,000 persons per year in Cameroon, and the risk of receiving a diagnosis of cancer before the age of 75 years was 8.7%. The age-standardized rate of cancer-related deaths was 73.1 per 100,000 persons per year, and the risk of dying of cancer before age 75 years was 11%^1^; the age-standardized incidence and mortality rates were 24 and 19 per 100,000 persons per year, respectively, for cervical cancer.^1^ Enow-Orock et al^2^ analyzed the Yaounde population-based cancer registry, and they found in 2005 that gynecologic cancers (GCs) had an incidence of 30 patients per month. Breast cancer was the most frequent GC (48.12%), and cervical cancer was the second most frequent (40.18%). In a retrospective study at the Yaounde Mother and Child Hospital, Sando et al^3^ found that cervical cancer represents 49.5% of GCs followed by breast cancer (34%) and ovarian cancer (7.4%). Although cervical cancer is a preventable disease, its incidence is increasing in our country because of poor-quality screening programs. The government is making an effort to detect and control infectious diseases such as HIV, tuberculosis, and malaria with free screening and treatment. The government in Cameroon, like those in other sub-Saharan countries, has not put as much effort into controlling cancer as it has controlling other noncommunicable diseases such as diabetes and hypertension.^1,4^ Thus, patients face difficulties in being screened and obtaining proper management of their disease. WHO and other authors have described determinants of poor cancer care, including barriers to accessing care centers, lack of financial means, transportation difficulties, and ignorance of risk factors.^5-7^ No studies have been conducted in Cameroon that evaluate the magnitude of and reasons for abandoning cancer care. Thus, to determine the reasons why patients abandon cancer care, our study was designed to evaluate the proportion of patients who leave cervical cancer care from the time their symptoms are clinically recognized to the time they receive curative treatment.

## PATIENTS AND METHODS

In January 2010, we initiated an in-hospital gynecologic cancer registry in the Limbe Regional Hospital to record patients’ personal information (address, phone number, e-mail address), sociodemographic information, histologic diagnosis, attitude toward care strategy, and patient’s goals. Data regarding cervical cancer were extracted from this registry, patients were observed prospectively via telephone calls, and additional information was obtained from referring physicians in other health care facilities.

Limbe is a cosmopolitan town in the South West Region of Cameroon, and its hospital is a regional reference hospital that serves a population of almost 1.5 million people. The region has no specialized structure dedicated to managing cancer (ie, no cytology or histology laboratories, cytology technicians, pathologists, or cancer care physicians) apart from one physician who was posted in 2015. Although easy screening techniques such as visual inspection using acetic acid or Lugol iodine were feasible, patients who required a biopsy were sent several miles out of the region or tissue specimens were transferred to other centers. For those diagnosed with cervical cancer, only two hospitals in the Cameroon cities of Douala and Yaounde offered a complete package of care (surgery, radiotherapy, and chemotherapy; radiotherapy equipment was functional only at the Douala Centre at the time). Only a few patients who were diagnosed early could be managed surgically at the regional level. The study included all patients whose symptoms suggested cervical cancer and who consented to participate. The study outcomes included the patient’s ability to pay for the biopsy and analysis and whether the patient accepted the diagnosis or not, came back to collect the results of histology tests, accepted the diagnosis, and visited the clinic regularly for the prescribed treatment. Answers to outcome questions were obtained directly from patients who attended follow-up appointments or via telephone. Data were analyzed using Epi Info version 3.5.3 software.

### Ethical Consideration

We sought and obtained ethics clearance from the Regional Hospital of Limbe institutional ethics committee. Counseling was offered to each patient included in the study, confidentiality of information was guaranteed, and verbal informed consent was obtained.

## RESULTS

**FIG 1:**
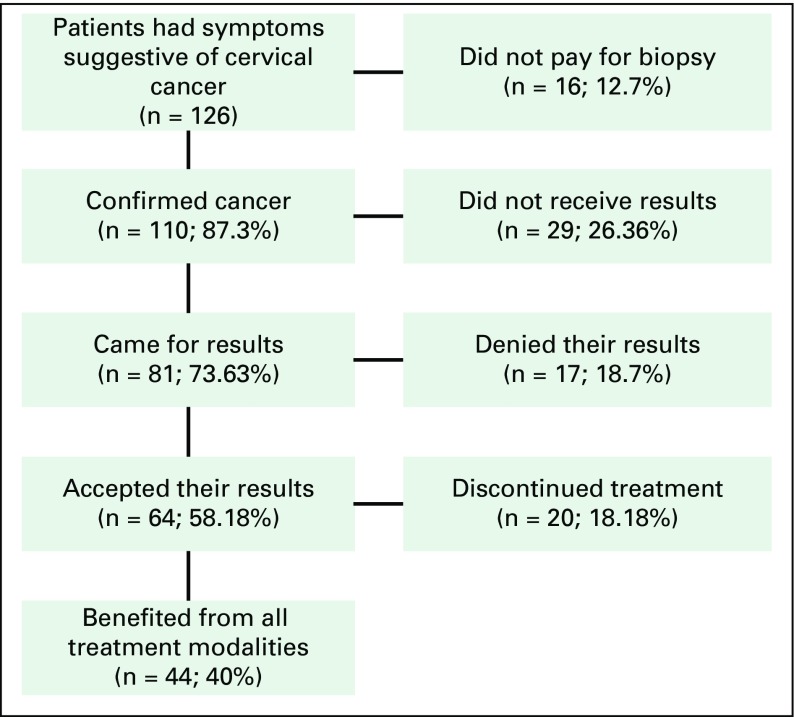
Compliance with management strategies.

During the study, 126 patients had symptoms suggestive of cervical cancer, but only 110 (87.30%) could pay for biopsy, 29 (26.36%) of those did not collect their results, 17 (18.7%) denied their results, and 20 (19%) did not benefit from treatment. From diagnosis to treatment, only 44 of 110 patients were able to complete their cancer care program; 60% were lost to follow-up (Fig 1).

Table 1 lists sociodemographic characteristics of study participants. Patients were 28 to 86 years old with a mean age of 51 ± 12 years. Fifty percent were in the 40-to-59-years-old age group (72.72% discontinued care); 78.19% were in the “not employed” category (which consisted of farmers, housewives, and household helpers; 84.10% were lost to follow-up); and civil servants represented 10.91% of the study participants (4.54% discontinued treatment). The majority of patients had primary (41.81%) or secondary (31.82%) education levels, and they represented the largest groups lost to follow-up with 34.1% and 31.81%, respectively.

**TABLE 1:**
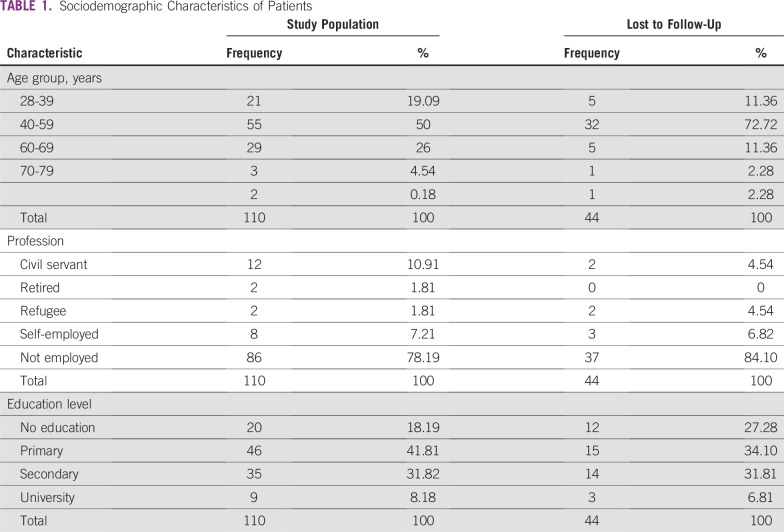
Sociodemographic Characteristics of Patients

### Compliance With Care Strategies

In all, 126 patients had symptoms suggestive of cervical cancer at presentation, but only 110 were able to pay for a biopsy, and those 110 were confirmed to have cancer. Of the 110 patients, 29 (26.36%) never returned to collect their results, and 17 (18.7%) returned to collect their results but denied having cancer. Sixty-four patients accepted their care plan but only 44 (40% of all patients with cancer) benefited from all of the proposed treatment modalities. Twenty patients (18.18%) left the care plan for various reasons (Fig 1).

## DISCUSSION

### 

The majority of patients diagnosed with cervical cancer in our study were in the 40-to-59-years-old age group (50%) or 28-to-39-years-old age group (19.09%), which is consistent with data from a study by Enow-Orock et al^2^ who found that 58% of patients with cervical cancer were age 34 to 58 years old. Tadesse et al^8^ and Bezabih et al^7^ in Ethiopia had 47% and 57% of patients in the 40-to-59-years-old age group. The greatest proportion of patients lost to follow-up (72.72%) were in that age group.

The majority (37 [84.10%] of 110) of those who discontinued treatment had no employment; (ie, they were small farmers, housewives, or household helpers with monthly incomes of about 32,000 FCFA (64 US dollars). An analysis of the cost of diagnosing cervical cancer in Cameroon showed that biopsies ranged from US $20 to US$40, which made it impossible for a disproportionate number of patients to be diagnosed. This situation has already been described by Echouffo-Tcheugui et al^1^ and other authors,^9,10^ who found that patients in low socioeconomic groups were also less adherent to screening programs and treatment.

Most of the patients in our study, like those in other studies,^11^ had a primary or secondary school level of education, and 34.10% and 31.81% were lost to follow-up, respectively. Mpiga et al^12^ in 2014 in Gabon found that the majority of patients who did not retrieve their colposcopy results had a university level of education. The difference in the findings could be explained by the fact that the Gabonese study was performed in an urban area, unlike our study, which was performed in a rural area.

### 

One hundred twenty-six patients had symptoms suggestive of cervical cancer but only 110 (87.3%) paid for biopsy. All of the 110 patients who paid for biopsy had their cervical cancer confirmed by histology; in fact, more that 90% of the patients had advanced stages (ie, stage II or above). Of the 110 patients, 29 (26.36%) never returned to collect their results. This was different from the findings by WHO in 2012, which reported that 65% of the 2,000 patients with cancer in seven developing countries failed to return for their results and treatment.^9^ The difference may be attributed to our small population sample. Reasons for not returning to collect results included the fear of having cancer (which a few patients considered a death sentence), and some patients could not afford to pursue the cancer management plan. Many authors describe lack of financial means to pay for treatment as the most important determinant of noncompliance with cancer care.^13-17^ Twenty (27%) of 64 patients who were willing to cooperate with their cancer care management plan finally discontinued treatment because of a lack of financial means to pay for it. Analysis of the financial situation of our study population showed that monthly income ranged from 32,000 FCFA (64 US$) for 56% of the patients to about 250,000 CFAF (500 US$) for less than 5% of the patients. But the cost of a course of chemotherapy is estimated to be roughly the same as the annual per-capita gross national product in Cameroon. This is the equivalent of patients with cancer in the United States paying approximately $47,000 for a course of generic chemotherapy.^10^ Other authors have identified absence of health coverage and lack of family support as likely determinants of treatment abandonment.^5^

Thirty percent of the 17 patients who denied their results, plus the 20 who could not adhere to their cancer care management plan claimed that they refused to be condemned to death or that “God would not let this happen to them.” In fact, they believed their illness was punishment from God and that only prayers or traditional healers could treat them. This attitude was described by Gerend and Pai^18^ in a study in the United States on social determinants of disparities among African American and white women; the former often have perceived invulnerability, folk beliefs, and a general mistrust of the health care system. Studies in Cameroon suggest that patients often believe their illness is caused by sorcery and witchcraft, so they resort to traditional healers or “men of God” rather than modern medicine.^10^ The proliferation of churches in which miraculous healing is preached is a real problem that needs to be addressed. Twenty percent of those who abandoned treatment said they did so because of their distance from the treatment center. In fact, the nearest treatment center (Douala) is 100 miles from Limbe, and many of our patients lived several hundred miles from Limbe and had to travel through areas with no roads. Our results are consistent with those of other authors who found that compliance with management strategies is driven by having a good health care delivery system.^1,19^

Our study highlights the magnitude of difficulties of cancer management in Cameroon. Only 35% of women whose symptoms suggested cervical cancer and 40% of those whose cancer was histologically confirmed remained under standard cancer care. Reasons for abandoning treatment include poverty in 27% of patients, belief in alternative treatment (according to religious beliefs or traditional healing) in 30% of patients, and a poor health care delivery system (lack of enough cancer care centers, reduced numbers of pathologists, and long distances from care centers). Redistribution of resources such as government subsidies and cancer care providers is mandatory if this situation is to improve.

## Data Availability

The following represents disclosure information provided by authors of this manuscript. All relationships are considered compensated. Relationships are self-held unless noted. I = Immediate Family Member, Inst = My Institution. For a detailed description of the disclosure categories, or for more information about ASCO’s conflict of interest policy, please refer to www.asco.org/rwc or ascopubs.org/jco/site/ifc. **Research Funding:** Pfizer **Travel, Accommodations, Expenses:** Laboratoire INNOTECH International **Research Funding:** Clinical Trials Unit, London School of Hygiene & Tropical Medicine No other potential conflicts of interest were reported.
